# Driving forces in amyloidosis: How does a light chain make a heavy heart?

**DOI:** 10.1016/j.jbc.2021.100785

**Published:** 2021-05-18

**Authors:** Daniel E. Otzen

**Affiliations:** iNANO, Aarhus University, Aarhus C, Denmark

**Keywords:** amyloidosis, light chains, variable domain, complementarity determining regions, clinical mutations, fibrillation, protein stability, protein dynamics, proteolysis, hydrogen–deuterium exchange MS, AL, light-chain amyloidosis, CDRs, complementary determining regions, C_L_, constant domain, HDX, hydrogen–deuterium exchange, Ig, immunoglobulin, LCs, light chains, V_L_, variable domain

## Abstract

Light-chain amyloidosis (AL) is a fatal disorder wherein the immunoglobulin light chain misfolds and aggregates, leading to amyloid plaques in various organs. Patient-specific mutations in the light chain variable domain (V_L_) are tightly linked to amyloidosis, but how these mutations drive AL is unknown. In recent work, Rottenaicher *et al.* analyze five mutations found in the V_L_ of a patient with cardiac AL. Their data suggest that decreased V_L_ stability and increased flexibility in the core of the V_L_, caused by mutations outside of this core, could be key to aggregation and highlight the delicate balancing act required for antibody maturation to enable antigen recognition while not altering protein biophysics.

Amyloidosis is a rare disorder caused by the misfolding of soluble amyloid protein, with the resulting aggregates interfering with organ function. Light-chain amyloidosis (AL) is the most common type of systemic amyloidosis and involves aggregation of fibrils formed by monomeric immunoglobulin (Ig) light chains (LCs), usually produced by malignant plasma cells (1). These aggregates can accumulate in various organs; the heart is a common target (2). The ~216-residue LC consists of two closely related Ig domains in tandem, the N-terminal variable domain (V_L_) and the constant domain (C_L_). Aggregates mostly consist of the V_L_, suggesting that the C_L_ has a stabilizing influence, which is lost upon proteolytic cleavage. However, cleavage alone is not sufficient to cause fibril formation. Various amyloidogenic mutations are involved, and many of these occur in complementarity-determining regions (CDRs) of the V_L_ (*i.e.*, the hypervariable segments that determine antigen selectivity) as a consequence of the natural mutagenesis that V_L_ undergoes during clonal selection. Moreover, CDRs not only bind antigens but also mediate contacts with the C_L_ and V_H_. Therefore, the introduction of inappropriate mutations represents a constant threat when combined with overproduction of LC. Solitary and ligand-free LCs are just looking for trouble! Given the wide variety of LCs and the many mutations that can be introduced, there is likely no universal explanation for AL, but each case story advances our understanding. A new case study from Rottenaicher *et al.* (3) examines the consequences of CDR mutations, demonstrating how a few seemingly small changes can have major impacts on protein stability and dynamics.

The native Ig fold of the V_L_ is as full of β-sheets as the amyloid fold adopted by the V_L_, but the molecule has to undergo a complete rearrangement to reach this alternative structure, which is prone to aggregate (4,5). Scientists have long struggled to understand how AL mutations lead to amyloidogenesis. There are many possible scenarios, but put simply, they involve push (weakening the native state by global or local destabilization), pull (stabilizing amyloidogenic states, including the end-state fibril), or both. As an extra dimension, the effect could also involve thermodynamics (a stable shift in populations), kinetics (decreased activation barriers to aggregates (6)), or both. Decades of research on protein structure and folding have led to the generalization that mutations to the hydrophobic core of a well-packed protein are the most destabilizing, and thus one would not expect loop residues to affect stability as much as framework mutations. However, the loop regions still have to be well packed to provide a stable binding surface for the selected antigen.

In recently published work, Rottenaicher *et al.* (3) dig deeper into an intriguing case of cardiac AL originally reported by Fändrich *et al.* (7). The patient’s V_L_ contains five mutations as compared with the deduced germline sequence (Fig. 1). Four are distributed among the three CDRs; of these, two are rather conservative changes (Y31S and N51S), whereas two others (G49R and G94A) would be classified by any protein engineer as disruptive introducers of new functional groups. The fifth mutation (a modest Y48F) is found right before CDR2. The relatively low number of mutations in this case made it straightforward for the researchers to dissect the impact of each change and ask how much change (*i.e.*, how many mutations) is required to stimulate fibril formation, what molecular properties of the proteins are affected by these mutations, and how are they linked with aggregation?

**Figure 1:**
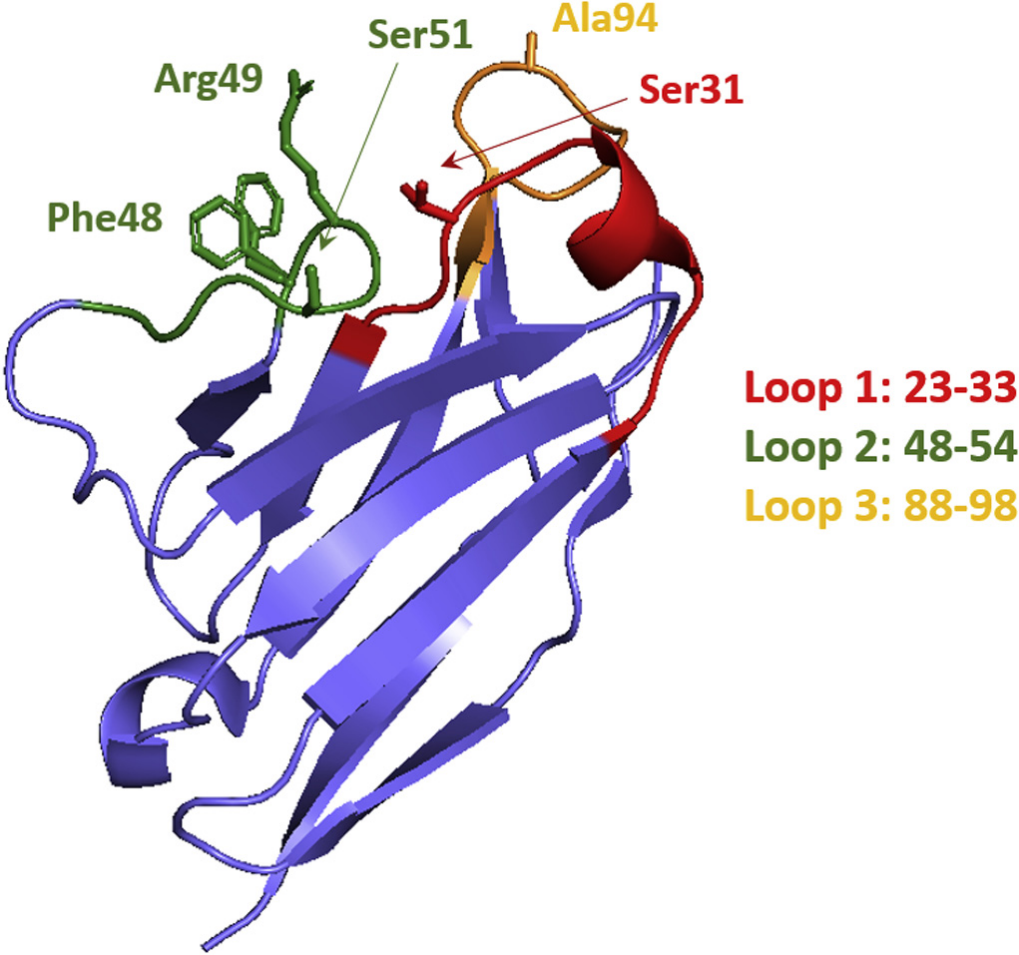
**A crystal structure of a V**_**L**_**monomer (PDB code 5L6Q) shows the relative location of the five residues unique to the patient sequence.** Two orientations of Phe48 and Ser31 are depicted. Three loops are depicted as follows: loop 1: residues 23 to 33 (*red*), loop 2: 48 to 54 (*green*), loop 3: 88 to 98 (*orange*). V_L_, variable domain.

To address these questions, the authors begin by comparing the available structure of this V_L_ mutant with a homology model of the parent structure. Although this comparison suggests there is no significant difference between the secondary, tertiary, or quaternary structures of the mutant and WT V_L_, the mutant aggregates after 4 days *in vitro*, whereas the WT remains stably folded over a 14-day period. Unsurprisingly, the mutant is significantly less stable than the WT, as measured by chemical and thermal denaturation. Five single mutants, each one of the mutations observed in the patient with AL, showed a spectrum of destabilizations, ranging from severe (G94A and G49R) and moderate (N51S) to fairly insignificant (Y48F and Y31S). Nevertheless, none of these mutations alone caused fibrillation, which was shown to occur only when G94A and G49R are combined in a double mutation. Indeed, this double mutant was nearly as unstable as the original patient sequence. Meanwhile, the slightly less unstable N51S/G94A double mutant fibrillates (albeit slowly) if helped along by a modest decrease of pH to 6.4. To test whether fibrillation is linked to more than just stability, the authors analyzed protein dynamics. Starting with the simple but illuminative test of resistance to proteolysis, they show that the more destabilized the protein is, the faster they are degraded on the global level. So far so good. However, when dynamics of the various mutants were probed at the local level (*i.e.*, the level of tryptic peptides), using hydrogen–deuterium exchange (HDX) MS, the authors stumbled upon a counterintuitive finding. HDX data taken after 2 h revealed an increase in dynamics in the framework (constant) region rather than the loops; that is, changes in the loop region are propagated to other parts of the protein. It would be intriguing to find out if earlier HDX time points would reveal more about the dynamics of the loops caused by mutations. Can we see the beginning of this structural unraveling around the sites of the mutations or do these loop residues just exchange fast in all cases? Finally, molecular dynamics simulations suggest that a reason for the destabilization by G49R and G94A is that these two residues are in regions of the Ramachandran plot, which are normally only favorable for Gly residues, and so, the mutations lead to highly stressed backbone conformations. The combined data lead the authors to speculate that increased flexibility leads to “the enhanced population of partially unfolded, aggregation-competent states.”

These intriguing results raise new questions about this patient sequence and protein folding more generally. It would be exciting to explore further how the loop mutations are transmitted to the framework region. Could one identify conditions (perhaps higher temperature combined with lowered pH) under which all of the mutants form fibrils, to be able to compare the stability and dynamics data with propensity to aggregate more generally? This might also reveal any possible aggregation-competent states whose existence currently eludes detection simply because of their low population and enable direct testing of the Reif group’s important conclusion that Arg49 directly stabilizes the fibril core (8). In contrast, G94A is thought only to destabilize the native state and not be important for fibril formation. Current models typically suggest that aggregation is simply proportional to level of destabilization, but the new results from Rottenaicher *et al.* put a renewed emphasis on the idea of shifts in the general ensemble of native-but-more-flexible states.

Where does this leave those afflicted with AL? Unlike other amyloidoses, all AL cases are (more or less) unique, so scientists and physicians trying to help patients with AL face extra challenges in trying to link molecular features with clinical outcomes. The ultimate goal would be to be able to predict aggregation propensity simply based on the sequence in combination with computational insight. While the Rottenaicher study reminds us how complicated proteins are, they also demonstrate that sound biophysical studies provide the insight to understand them better. Perhaps, a concerted effort to study a large group of different LC cases worldwide using these approaches would allow us to build a robust and predictive model that can deliver “diagnoses while you wait” in the not-too-distant future. The next step is to do something about the aggregation problem—but that is another story.

## Conflict of interest

The author declares that he has no conflicts of interest with the contents of this article.
